# Hippocampal Infusion of Zeta Inhibitory Peptide Impairs Recent, but Not Remote, Recognition Memory in Rats

**DOI:** 10.1155/2015/847136

**Published:** 2015-08-24

**Authors:** Jena B. Hales, Amber C. Ocampo, Nicola J. Broadbent, Robert E. Clark

**Affiliations:** ^1^Department of Psychiatry, University of California, San Diego, La Jolla, CA 92093, USA; ^2^Department of Psychology, University of California, San Diego, La Jolla, CA 92093, USA; ^3^Veterans Affairs San Diego Healthcare System, San Diego, CA 92161, USA

## Abstract

Spatial memory in rodents can be erased following the infusion of zeta inhibitory peptide (ZIP) into the dorsal hippocampus via indwelling guide cannulas. It is believed that ZIP impairs spatial memory by reversing established late-phase long-term potentiation (LTP). However, it is unclear whether other forms of hippocampus-dependent memory, such as recognition memory, are also supported by hippocampal LTP. In the current study, we tested recognition memory in rats following hippocampal ZIP infusion. In order to combat the limited targeting of infusions via cannula, we implemented a stereotaxic approach for infusing ZIP throughout the dorsal, intermediate, and ventral hippocampus. Rats infused with ZIP 3–7 days after training on the novel object recognition task exhibited impaired object recognition memory compared to control rats (those infused with aCSF). In contrast, rats infused with ZIP 1 month after training performed similar to control rats. The ability to form new memories after ZIP infusions remained intact. We suggest that enhanced recognition memory for recent events is supported by hippocampal LTP, which can be reversed by hippocampal ZIP infusion.

## 1. Introduction

Several reports have now demonstrated that spatial memory can be erased by infusing zeta inhibitory peptide (ZIP), a cell-permeable synthetic peptide, into the dorsal hippocampus [1–3]. In these studies, ZIP was infused into the dorsal hippocampus after rodents were trained on a spatial task. When the animals were tested following ZIP infusion, there was no evidence of memory retention—the memories appeared to have been erased. ZIP is thought to erase spatial memory by reversing established late-phase long-term potentiation (LTP). LTP is a function of enhanced AMPA receptor-mediated transmission at potentiated synapses, and ZIP is thought to interrupt the intercellular signaling pathways that traffic and maintain AMPA receptors at the postsynaptic density [4]. Such findings are important because they add to a substantial literature showing that the hippocampus is critical for spatial memory. In addition, these findings extend prior work by indicating that LTP (and perhaps PKMzeta; see [5]) is the physiological mechanism that supports long-term spatial memory. At present it is unclear whether hippocampal LTP also supports other forms of hippocampus-dependent memory, such as recognition memory.

Recognition memory is the ability to judge a previously encountered item as familiar and is dependent on structures in the medial temporal lobe (MTL) [6], including the hippocampus [7]. Recognition memory is a pervasive and critical form of memory which is most commonly tested in the experimental animal with the novel object recognition (NOR) task. For anterograde memory, the NOR task has proven to be sensitive to hippocampal damage or disruption in humans [8, 9], monkeys [10–12], rats (e.g., [13]), and mice (e.g., [14]). The NOR task is also sensitive to hippocampal damage when the damage occurs after the learning episode [15, 16]. At present, only a single study has examined recognition memory following hippocampal infusion of ZIP. In this case, ZIP infusion did not impair object recognition memory, although the infusion did impair the spatial version of this task [3], suggesting that object recognition memory is supported by a different physiological mechanism than spatial memory. However, another possibility is that object recognition memory was unaffected because an insufficient area of the hippocampus was disrupted by the infusion. In the Hardt et al. [3] study, as well as in all other studies that have infused ZIP into the hippocampus, only the dorsal aspect of the hippocampus was targeted. This is because the standard method is to implant bilateral indwelling guide cannulas into the hippocampus so that ZIP can be infused after the training episode. This method only allows the dorsal aspects of the hippocampus to be reached while sparing the entire ventral portion of the hippocampus. Importantly, prior work has shown that while dorsal hippocampal damage is sufficient to impair spatial memory [17–19], both the dorsal and ventral regions of the hippocampus must be damaged in order to produce object recognition memory impairments [19].

In our study, we circumvented the restriction of the cannulation method by exploiting a unique feature of compounds that are able to reverse late-phase LTP. Unlike pharmacological compounds which must be infused either immediately before, during, or immediately after the learning episode (e.g., [20]), compounds like ZIP are able to impair memory even days after the learning episode [3, 21]. The fact that ZIP can be infused even days after the learning episode obviates the need for indwelling guide cannulas. Instead, animals can be trained and then ZIP can be infused the next day, or later, using an infusion needle during stereotaxic surgery. This stereotaxic approach allows ZIP to be infused at any number of precisely targeted locations. In this study, following training on the NOR task, rats underwent stereotaxic surgery and ZIP was infused into all regions of the dorsal, intermediate, and ventral hippocampus. Because prior work has demonstrated that recognition memory starts out as being hippocampus-dependent but becomes hippocampus-independent during the weeks after learning [16], we examined how reversing LTP with ZIP infusions affected both recent memory (3–7 days old) and remote memory (1 month old).

## 2. Methods

### 2.1. Subjects

All experimental procedures were approved by the Institutional Animal Care and Use Committee at the University of California, San Diego. Subjects were 86 male, Long-Evans rats weighing between 300 and 350 g at the beginning of the study. Rats were individually housed and maintained on a 12 : 12 h light : dark cycle. Food and water were freely available. Rats were randomly assigned to receive bilateral infusions of ZIP or aCSF 3–7 days after training (ZIP recent, *n* = 10; aCSF recent, *n* = 10) or 32–36 days after training (ZIP remote, *n* = 24; aCSF remote, *n* = 24). One aCSF remote rat was excluded from the test analysis due to an error with data collection. An additional group of control rats that did not undergo surgery were included for recent memory testing (control recent, *n* = 16). Two additional rats were used for immunohistological assessment of the extent of the ZIP infusion.

### 2.2. Apparatus

The novel object recognition task was conducted in an opaque plastic box measuring 35 cm × 41.5 cm × 50 cm. Stimuli consisted of ceramic or plastic objects that varied in color and size (see [16] for details).

### 2.3. Habituation and Familiarization

Rats were acclimated to the testing room and habituated to the empty box for five min each day for two days. Rats then had 4 days of familiarization during which they were placed in the box for 15 min per day and allowed to explore two identical objects. Each rat had the same objects during every familiarization day, and the specific object was counterbalanced across rats. Following familiarization, rats were divided into ZIP and aCSF infusion groups. Rats underwent surgery 3–7 days or 32–36 days after training.

### 2.4. Surgery

Anesthesia was maintained throughout surgery with isoflurane gas (0.8%–2.0% isoflurane delivered in O_2_ at 1 L/min). The rat was placed in a Kopf stereotaxic instrument, and the incisor bar was adjusted until the dorsal surface of the skull was level. ZIP or aCSF was infused bilaterally throughout the hippocampus with a 10 *μ*L Hamilton syringe mounted on a stereotaxic frame and held with a Kopf Microinjector (model 5000). Biotinylated ZIP (Tocris Bioscience; Ellisville, Missouri) (1 mg) was reconstituted in 100 *μ*L of sterile water with a resulting stock solution concentration of 10 *μ*M ZIP. 10 *μ*L of the 10 *μ*M ZIP was then diluted in 9.99 mL of aCSF to provide a solution with a concentration of 10 nM ZIP/1 *μ*L aCSF. The syringe needle was lowered to the target coordinate and left in place for 1 min before beginning the injection. A total of 4.8 *μ*L of ZIP or aCSF was injected into 8 sites within each hippocampus. All coordinates are in millimeters anteroposterior (AP) and dorsoventral (DV) relative to Bregma and mediolateral (ML) relative to Lambda: AP −2.8, ML ±2.2, DV −3.8; AP −3.8, ML ±3.4, DV −3.6; AP −4.8, ML ±3.4, DV −3.8; AP −4.8, ML ±5, DV −8.4, −5; AP −5.6, ML ±4.8, DV −8, −6, and −4. Once awake and responsive, each rat was returned to its home cage for a 5–8-day recovery period.

### 2.5. Test

After recovering from surgery, rats were returned to the testing box and allowed to explore two objects (one novel object and a copy of the object from the familiarization phase) for 15 minutes. Using video recordings, object exploration was scored when a rat's nose was within 1 cm of the object and the vibrissae were moving (see [13]). Object exploration was not scored when the rat reared upwards facing the ceiling or leaned on the object. Object recognition memory was inferred by a preference for the novel object compared to the familiar and thus less interesting object. The time spent exploring the novel object was divided by the time spent exploring the novel object + the time spent exploring the familiar object. This value was then multiplied by 100 (chance performance = 50%; see [16] for more details).

### 2.6. New Learning

After completing the retention test, rats in the remote memory group were given a new NOR trial. Rats were placed in the box for a 15 min familiarization phase and allowed to explore two new and identical objects. Following a 3 h delay period, during which rats remained in the testing room in their home cages, rats were returned to the box with two objects (one novel object and a copy of the object from familiarization). Object exploration was scored and analyzed as described above.

### 2.7. Histology

At completion of testing, the rats were administered an overdose of sodium pentobarbital and perfused transcardially with buffered 0.9% NaCl solution followed by 10% formaldehyde solution (in 0.1 M phosphate buffer). The brains were then removed and cryoprotected in 20% glycerol/10% formaldehyde. Coronal sections (50 *μ*m) were cut with a freezing microtome ranging from the anterior commissure through the length of the hippocampus. Every third section was mounted and stained with thionin to assess the position of the needle track and any unintended damage. Each section was assessed under magnification.

Two additional rats were perfused with 1x phosphate-buffered saline (PBS) and 4% paraformaldehyde solution two hours after ZIP infusion to visualize the extent of the ZIP infusion. Brains were removed and stored in 4% paraformaldehyde overnight at 4°C and transferred to 1x PBS solution. Coronal sections (40 *μ*m) were cut as described above; however, every fifth section was stained to visualize the spread of the infused biotinylated ZIP. A mouse anti-biotin primary antibody (Jackson Immuno, 200-002-211, 1 : 400) and a fluorescent donkey anti-mouse Cy3 secondary antibody (Jackson Immuno, 715-165-150, 1 : 100) were used, along with DAPI (1 : 1000) as a counterstain for cell bodies. An additional series of sections was mounted and stained with thionin to visualize the hippocampal cell layers.

## 3. Results

### 3.1. Histology

**Figure 1:**
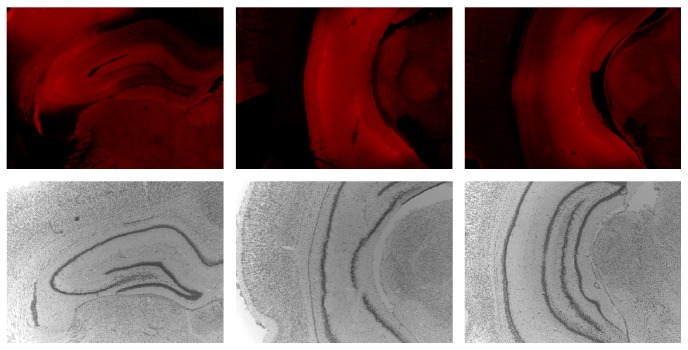
Photographs at three coronal levels for a rat with hippocampal infusion of ZIP (anterior to posterior from left to right; lateral to the left of each section and medial to the right). Top: fluorescence images depicting the extent of the ZIP infusion throughout all cell layers of dorsal, intermediate, and ventral hippocampus, while remaining confined to the hippocampus. Some sparing was noted in the most medial aspects of the dorsal hippocampus. The only biotin-labeled ZIP outside of the hippocampus was the result of diffusion along the needle track. Bottom: corresponding tissue stained with thionin to visualize the hippocampal cell layers.

Figure 1 depicts the extent of the ZIP infusion throughout the hippocampus. ZIP infusion covered all cell layers of dorsal, intermediate, and ventral hippocampus and was confined to the hippocampus. Some sparing was noted in the most medial aspects of the dorsal hippocampus. The only biotin-labeled ZIP outside of the hippocampus was the result of diffusion along the needle track.

### 3.2. Behavior

**Figure 2:**
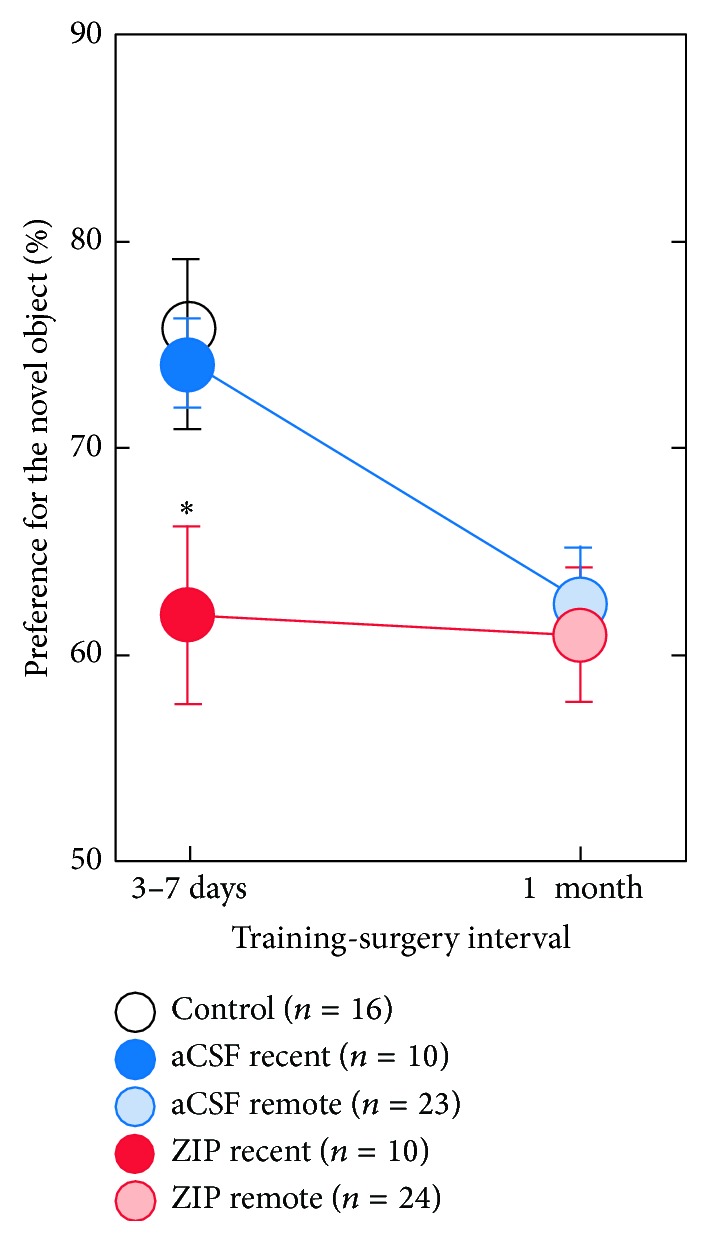
Preference for the novel object after infusion for recent and remote memory conditions. In the recent memory condition, all groups performed above chance, but rats with ZIP infusions (red) performed worse than rats with aCSF infusions (blue) and control (white) rats. In the remote memory condition, both ZIP (light red) and aCSF (light blue) groups performed similarly and above chance. Error bars indicate SEM. Asterisk indicates difference from aCSF and control groups (*P* < 0.05). All groups performed above chance (*P* < 0.05, chance = 50%).

In the recent memory condition, all groups performed above chance (ZIP: *t*_(9)_ = 2.78, *P* < 0.05; aCSF: *t*_(9)_ = 7.31, *P* < 0.0001; control: *t*_(15)_ = 8.37, *P* < 0.0001), but rats with ZIP infusions performed worse than rats with aCSF infusions (*t*_(18)_ = 2.27, *P* < 0.05) and control rats (*t*_(24)_ = 2.72, *P* < 0.05). Rats with aCSF infusions and control rats, however, performed similarly (*t*_(24)_ = 0.39, *P* > 0.1). In the remote memory condition, both ZIP and aCSF groups performed similarly (*t*_(45)_ = 0.39, *P* > 0.1) and above chance (ZIP: *t*_(23)_ = 3.42, *P* < 0.01; aCSF: *t*_(22)_ = 4.89, *P* < 0.0001) (Figure 2).

**Figure 3:**
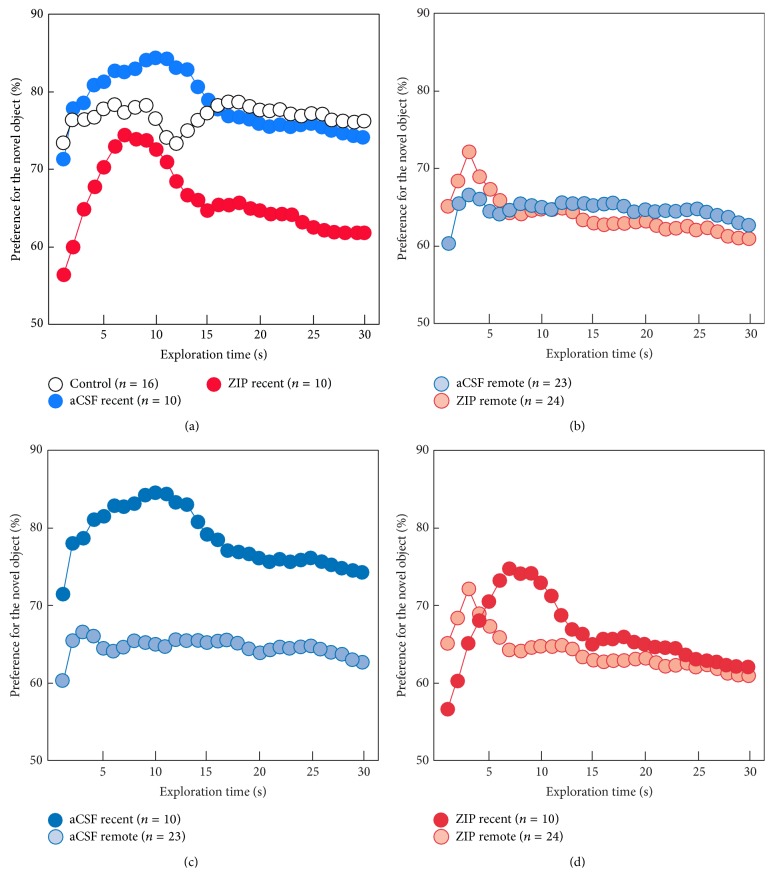
Cumulative percent preference for the novel objects across 30 sec of object exploration for the three groups in the recent condition (a), the two groups in the remote condition (b), the recent and remote aCSF groups (c), and the recent and remote ZIP groups (d). The pattern of performance indicates that the aCSF recent group and the unoperated control group showed a stronger preference for the novel object across the entire 30 sec test than the ZIP recent, ZIP remote, or aCSF remote group.

Figure 3 shows the cumulative percent preference for the novel objects across 30 sec of object exploration for each group in each condition. The pattern of performance indicates that the aCSF recent group and the control group showed a stronger preference for the novel object across the entire 30 sec test than the ZIP recent, ZIP remote, or aCSF remote group. Rats that received aCSF infusions 3–7 days after training and testing 1 week after surgery performed better than rats that received aCSF infusions 1 month after training and testing 1 week after surgery (Figure 3(c); at 30 sec of object exploration: *t*_(31)_ = 2.57, *P* < 0.05). However, rats that received ZIP infusions 3–7 days after training and testing 1 week after surgery performed similar to rats that received ZIP infusions 1 month after training and testing 1 week after surgery (Figure 3(d); at 30 sec of object exploration: *t*_(32)_ = 0.16, *P* > 0.1).

### 3.3. Retraining

**Figure 4:**
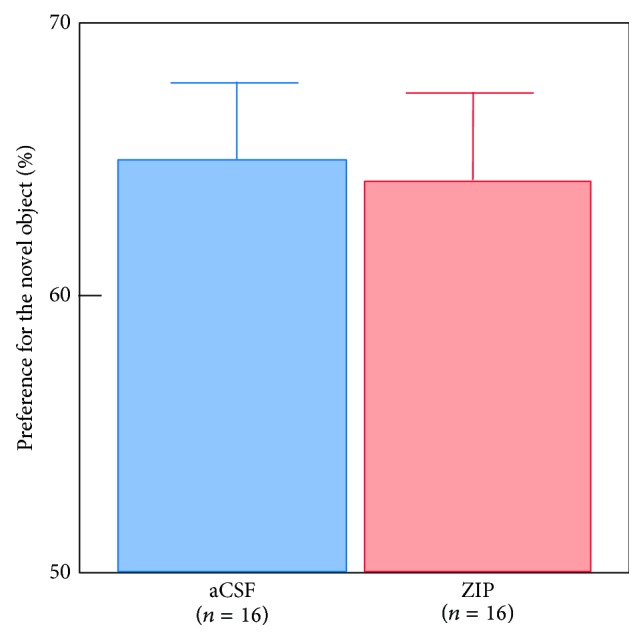
On the test of new learning, ZIP remote rats (light red) performed similar to aCSF remote rats (light blue) and both groups performed above chance level (chance = 50%). These data indicate intact learning after ZIP infusion.

On the test of new learning, ZIP remote rats performed similar to aCSF remote rats (*t*_(30)_ = 0.17, *P* > 0.1), and both groups performed above chance (ZIP: *t*_(15)_ = 4.52, *P* < 0.001; aCSF: *t*_(15)_ = 5.24, *P* < 0.0001) (Figure 4). These data indicate that, after infusion of ZIP, rats are not impaired at learning new objects.

## 4. Discussion

Rats were given a single 15 min familiarization phase on each of 4 consecutive days before receiving either bilateral infusions of ZIP or aCSF (or serving as unoperated controls) into the dorsal, intermediate, and ventral regions of the hippocampus during stereotaxic surgery. The surgeries were conducted 3–7 days (recent group) or 1 month (remote group) after the final familiarization day. On a retention test approximately 1 week after surgery, the recent ZIP group exhibited impaired object recognition memory compared to the aCSF and unoperated control groups. In contrast, the remote ZIP group performed similar to the remote aCSF group (Figures 2, 3(a), and 3(b)). All groups in both recent and remote conditions performed better than chance. These data indicate that only recent memory is susceptible to ZIP infusion. Finally, when the remote ZIP and remote aCSF groups were given a new NOR trial and tested with a 3-hour delay, both groups performed similarly and above chance. These data indicate that ZIP infusion did not disrupt the animal's ability to form new recognition memories.

These data add to a growing literature that indicates hippocampus-dependent memory can be disrupted by reversing late-phase hippocampal LTP by the infusion of zeta inhibitory peptide, ZIP. ZIP is thought to reverse late-phase LTP and impair memory by inactivating PKMzeta (for review see [4, 5, 22]). While there is some dispute concerning the relationship of ZIP and PKMzeta (for review see [23]), PKMzeta is thought to maintain LTP by persistently upregulating AMPA receptor trafficking for insertion into postsynaptic sites [24]. PKMzeta, an atypical isoform of protein kinase C, is unique in that it does not contain a regulatory region. Thus, once synthesized, PKMzeta remains constitutively active without requiring second messenger binding. It is believed that this particular feature of PKMzeta allows it to actively maintain the facilitated synaptic connections that represent long-term memory [4, 25]. We suggest that PKMzeta may be the molecular mechanism for maintaining the enhanced portion of recent object recognition memory.

This is the first study to show that object recognition memory can be disrupted by ZIP infusion into the hippocampus. Prior work reported that ZIP infusion into the dorsal aspect of the hippocampus was sufficient to impair a spatial version of the NOR task where one of two identical objects is physically relocated to a different part of the testing box [3]. However, in the same study, object recognition memory, as measured by the NOR task, was entirely unaffected. A critical difference between that study and the present study is that we infused ZIP into the dorsal, intermediate, and ventral aspects of the hippocampus, whereas Hardt et al. [3] targeted only the dorsal hippocampus. The findings from these two ZIP studies ([3] and the current study) are consistent with studies that have used permanent hippocampal lesions to study spatial and object recognition memory. For example, rats with hippocampal lesions exhibited impaired spatial memory for the location of a hidden platform in the water maze when approximately 30–50% of the dorsal hippocampus was damaged [17–19]. Increasing the amount of damage beyond 50% did not exacerbate the deficit. Importantly, for object recognition memory, only nearly complete lesions of the dorsal and ventral hippocampus (75–100%) were sufficient to impair performance [19]. Furthermore, that study found that dorsal or ventral lesions alone impaired spatial memory, but not object recognition memory, which required 75–100% of the entire hippocampus to be damaged. As there are no obvious anatomical or physiological characteristics of the ventral hippocampus that would explain why the object recognition deficit results from extending the dorsal lesion to include the ventral hippocampus, we suggest that the impairment results from a more complete disruption of hippocampal function. The current study has added advantages of using ZIP infusions over neurotoxic lesions, namely, that the hippocampus is still intact during the test for retrograde memory, thereby avoiding the confounding of a potential performance deficit due to nonmnemonic functions of the hippocampus. ZIP infusion during stereotaxic surgery also makes it possible to target the entire hippocampus, which is not possible with pharmacological infusions requiring an implanted guide cannula.

An important difference between the present finding of impaired object recognition memory following hippocampal ZIP infusion and prior work where ZIP was infused after spatial learning is that, in tests of spatial memory, ZIP appeared to erase memory. That is, spatial memory was completely eliminated with no evidence of recovery [1–3]. In this study, object recognition was only impaired in the recent condition relative to the aCSF and unoperated control groups and, importantly, the ZIP groups performed better than chance on both the recent and remote conditions (Figure 3(d)). Following complete hippocampal lesions, we have previously reported a temporally graded NOR impairment [16]. In contrast, Gaskin et al. [15] found both recent and remote retrograde impairment; however, their findings are complicated by the fact that the remote control group did not perform better than chance (i.e., *P* < 0.05), which would have made observing a temporal gradient in the control group unlikely. In the current study, ZIP infused rats performed above chance at both recent and remote time points, but they were impaired relative to the control groups only in the recent condition. Therefore, these results support differential involvement of the hippocampus in recent and remote object recognition memory, but the differences between permanent neurotoxic lesions and ZIP infusions must be appreciated when comparing such findings. Figure 3 is presented to provide a more thorough visualization of the behavioral phenotypes for the recent and remote ZIP and aCSF group.  Figure 3(a) shows the strong and robust memory performance of the two control groups for the recent condition. The ZIP group, while performing better than chance, exhibited weaker memory performance than the two control groups.  Figure 3(b) shows the nearly identical performance of the ZIP and aCSF groups on the remote memory condition. Figure 3(c) clearly illustrates how much stronger the memory was in the recent aCSF group compared to the remote aCSF group. Finally, Figure 3(d) shows how similar the performance was between the recent and remote ZIP groups. Taken together, these data indicate that object recognition memory* per se* was not dependent on hippocampal LTP. Rather, only the enhanced portion of the memory exhibited by the control groups in the recent condition was hippocampal LTP-dependent. ZIP infusion in the recent condition has the effect of turning a strong object recognition memory into a weak recognition memory. This appears to be very similar to what happens naturally as strong recent memory becomes weak remote memory (Figure 3(c)). Accordingly, infusing ZIP in the remote condition had no appreciable effect on memory, presumably because the LTP-dependent, enhanced portion of memory seen in the recent condition had faded away.

Recognition memory is typically described as consisting of two components, most often referred to as familiarity and recollection [26]. Familiarity consists of only knowing that an item has been previously encountered. In contrast, recollection includes recalling specific contextual information that accompanied the specific learning episode. Theoretical accounts of this distinction have suggested that different brain structures independently support these two components, with the perirhinal cortex supporting familiarity-based recognition memory and the hippocampus supporting recollection-based recognition memory [27–29]. However, because the NOR task can be accomplished solely by familiarity-based memory, examples of hippocampal damage impairing performance on the NOR task count against this idea. While examples in rats can be found where hippocampal lesions do not impair performance on the NOR task (for review see [30]), there are many examples of impaired performance on the NOR task in humans [8, 9], monkeys [10–12], rats (e.g., [13]), mice (e.g., [14]), and the current study (also, see [7] for review). The anatomical basis of recognition memory has recently been reconceptualized, drawing on human fMRI studies, studies of amnesic patients, monkey physiology work, and rodent lesion studies [6]. Here, the authors suggest that the perirhinal cortex and the hippocampus can be better understood as working together to accomplish both familiarity and recollection-based recognition memory. Importantly, the authors propose that hippocampal activity is particularly important for forming strong recognition memories (for both familiarity and recollection-based memory). Taken together with the current findings, we suggest that the perirhinal cortex can, to a limited extent, support recognition memory in the absence of the hippocampus, but robust, strong recognition memory requires the hippocampus and hippocampal LTP.
